# Effects of Ethanol on the Expression Level of Various BDNF mRNA Isoforms and Their Encoded Protein in the Hippocampus of Adult and Embryonic Rats

**DOI:** 10.3390/ijms161226242

**Published:** 2015-12-21

**Authors:** Shahla Shojaei, Saeid Ghavami, Mohammad Reza Panjehshahin, Ali Akbar Owji

**Affiliations:** 1 Department of Biochemistry and Recombinant Protein Laboratory, School of Medicine, Shiraz University of Medical Sciences, Shiraz 713484579, Iran; shahla_shojaei@yahoo.com; 2 Department of Human Anatomy and Cell Science, Faculty of Health Sciences College of Medicine, University of Manitoba, Winnipeg, MB R3E 0J9, Canada; saeid.ghavami@umanitoba.ca; 3 Health Research Policy Centre, Shiraz University of Medical Sciences, Shiraz 713484579, Iran; 4 Department of Pharmacology, School of Medicine, Shiraz University of Medical Sciences, Shiraz 713484579, Iran; panjeshm@sums.ac.ir; 5 Research Center for Psychiatry and Behavioral Sciences, Shiraz University of Medical Sciences, Shiraz 713484579, Iran

**Keywords:** alcohol, neuroprotection, neurotoxicity, growth factor, resveratrol, fetus, transcript

## Abstract

We aimed to compare the effects of oral ethanol (Eth) alone or combined with the phytoestrogen resveratrol (Rsv) on the expression of various brain-derived neurotrophic factor (BDNF) transcripts and the encoded protein pro-BDNF in the hippocampus of pregnant and embryonic rats. A low (0.25 g/kg body weight (BW)/day) dose of Eth produced an increase in the expression of BDNF exons I, III and IV and a decrease in that of the exon IX in embryos, but failed to affect BDNF transcript and pro-BDNF protein expression in adults. However, co-administration of Eth 0.25 g/kg·BW/day and Rsv led to increased expression of BDNF exons I, III and IV and to a small but significant increase in the level of pro-BDNF protein in maternal rats. A high (2.5 g/kg·BW/day) dose of Eth increased the expression of BDNF exons III and IV in embryos, but it decreased the expression of exon IX containing BDNF mRNAs in the maternal rats. While the high dose of Eth alone reduced the level of pro-BDNF in adults, it failed to change the levels of pro-BDNF in embryos. Eth differentially affects the expression pattern of BDNF transcripts and levels of pro-BDNF in the hippocampus of both adult and embryonic rats.

## 1. Introduction

Ethanol (Eth) consumption is known to cause deleterious effects on adults and the developing brain [1,2,3]. Long-term alcohol intake by adult humans is associated with cerebellar atrophy and disturbed neuronal function within the hippocampus and frontal cortex [4]. Rats also show impairment of both motor function and cognition following Eth consumption [5]. In addition to its neurodegenerative effects on the adult central nervous system (CNS), Eth may affect the fetal CNS structurally and functionally if it is abused during pregnancy (reviewed in [6]). This will lead to impairment of cognitive functions, such as learning and memory, and of facial features that are hallmarks of fetal alcohol syndrome [7]. Results of many investigations show that rodents exposed prenatally to Eth also show many features of human fetal alcohol syndrome [8,9]. Many of these features result at least partly from aberrant effects of Eth on the hippocampus [10]. Notably, this tissue mediates memory and cognition that are impaired in alcoholics and, thus, Eth-induced neurotoxicity has been substantially studied in the hippocampus [11,12].

Extensive research has been performed to determine the mechanism of Eth-induced neurodegeneration. One early suggestion pointed to the neuroapoptosis resulting from hyper-activation of γ amino butyric acid (GABA) A receptors and from inhibition of *N*-methyl-d-aspartate (NMDA) glutamate receptors [13]. Other proposed mechanisms include disturbance of potassium channel currents [14], induction of oxidative stress [15], modulation of retinoic acid signaling [16], thiamine deficiency [17], disruption of translational regulation and interference with signaling by neurotrophic factors [18]. There is also much evidence that some of the teratogenic manifestations observed in rats that are prenatally exposed to Eth are related to impaired neurotrophin function and expression [19]. In this context, Eth-induced disturbances in the signaling of brain-derived neurotrophic factor (BDNF) is the most reported because of the high impact of this neurotrophin on learning and memory in adulthood, and its effects on neural development and cognition in childhood [20,21].

The rat BDNF gene has a complicated structure, consisting of eight noncoding exons (I–VIII) in the 5′ untranslated region (UTR), each with its own promoter and a common coding exon (IX) in the 3′ region that contains the entire open reading frame of the BDNF protein. This gene is transcribed to 11 primary transcripts each characterized by one 5′ UTR exon linked by alternative splicing to the common coding exon IX. All 11 distinct mature BDNF mRNAs are translated into an identical pro-BDNF protein [22]. The site and the exact enzyme for proteolytic processing of pro-BDNF to BDNF is a matter of debate [23]. However, in addition to being a precursor for BDNF, pro-BDNF has shown to act as a signaling molecule through distinct receptors [24]. It has been shown that BDNF and pro-BDNF increase and decrease neuronal cell survival through separate receptors [25,26].

UTRs have shown established roles in the regulation and stability of transcription in addition to modulation of translation initiation and efficiency [27,28]. However, additional roles have been proposed for various 5′ UTRs of the BDNF gene, such as differential expression in different parts of the CNS during life-span [22,29]. In addition, the 5′ UTR of BDNF mRNAs is suggested to be important in mRNA localization in distinct neuronal compartments such as soma and proximal or distal dendrites [30].

Resveratrol (Rsv) is known as an effective neuroprotective factor, a property that may be related to its broad spectrum of beneficial effects [31,32,33]. Some investigators have attributed the neuroprotective effects of Rsv to its potent antioxidant properties [34]. Additionally, Rsv can be neuroprotective through its anti-apoptotic properties, which are shown to be linked to its activating effects on sirtuins [35,36,37] or via its inducing effects on BDNF mRNA levels [38,39]. Rsv can be easily absorbed when taken orally [40] and pass through the blood-brain barrier [32] and the placenta [41].

The splice variants of the BDNF gene are transcribed independently of each other [22]. Various treatments have shown a modulatory effect on the expression levels of different BDNF splice variants with a profile that depends on the nature of the treatment [13,42,43,44]. In the present study, we aimed to compare the effects of low and moderate doses of Eth on the expression profile of 5′ UTR exons I, III, IV and IX of the BDNF gene and its encoded protein pro-BDNF in the hippocampi from adult and embryonic rats.

## 2. Results and Discussion

### 2.1. Effects of Eethanol on the Expression Pattern of Exons I, III, IV and IX of the BDNF Gene in the Hippocampus of Adult and Embryonic Rats

Based on WHO reports, females are more sensitive to deleterious effects of Eth than males [45]. However, there is little data on the effect of Eth consumption on BDNF gene expression at either the mRNA or protein levels in female rats. Despite established neurotoxic effects of Eth on the fetus [8,46,47], there is no information on the effect of Eth on BDNF gene expression levels in the embryonic rats. However, results of numerous studies have reported that BDNF and its signaling pathway molecules are related to Eth neurotoxicity [21,48,49,50]. Eth effects on BDNF expression have been studied in the hippocampus of male and neonatal rats and in the hippocampal cells in culture, but the results are not consistent. While data from some studies show that Eth caused a decrease [51,52] and/or no change [53,54] in hippocampal BDNF levels, results from other studies show an Eth-induced increase in BDNF transcript expression levels in cultured hippocampal pyramidal cells [55].

**Figure 1:**
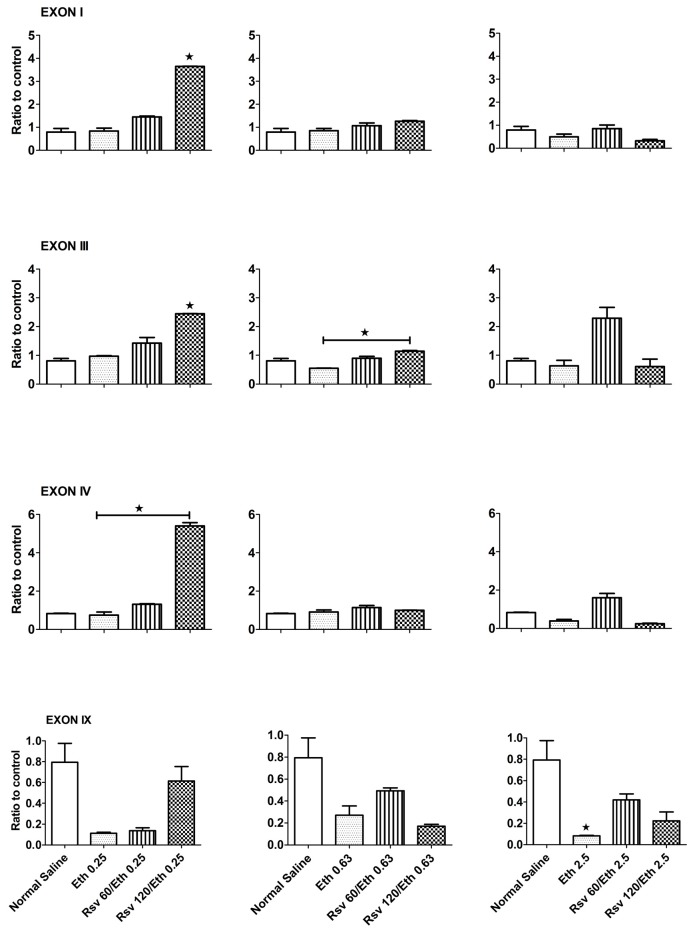
Eth and Rsv affect brain-derived neurotrophic factor (BDNF) exon expression in the hippocampus of pregnant rats differently. Eth had no significant effect on expression of any of the BDNF exons except at a dose of 2.5 g/kg·BW/day, which caused a decreased in the expression of BDNF exon IX (*p* < 0.05). Higher doses of Rsv in combination with Eth (0.25 g/kg·BW/day) significantly increased the expression of the BDNF exons I and III compared to the normal saline group and also increased expression of the BDNF exon IV compared to the Eth group (*p* < 0.05). Rsv in combination with Eth (0.63 g/kg·BW/day) significantly increased expression of BDNF exon III compared to the Eth (0.63 g/kg·BW/day) group (*p* < 0.05). Rsv in combination with Eth (2.5 g/kg·BW/day) reversed the significant decrease in the expression of BDNF exon IX at higher doses (*p* < 0.05), while it had no effect on the expression of other BDNF exons. qRT-PCR analysis was performed to evaluate expression of BDNF transcripts containing exons I, III, IV and IX in the hippocampi of pregnant rats. Rats were treated by either Eth alone or in combination with RSV using oral gavage for 20 days, from day 1 to 20 of gestation, and another group was treated with normal saline as a control group (Table 1). The right hippocampi from four pregnant rats were pooled to make one sample (three samples in each group, for a total of 12 hippocampi). Expression of β-actin was analyzed to normalize gene expression in each sample to eliminate differences in RNA quality and amplification efficacy. Data are presented as the fold increase in the expression in treatment groups compared to the control group, and are presented as the mean ± standard error of the mean (SEM). * *p* < 0.05

We used real-time polymerase chain reaction (PCR) to assess the expression of mRNAs containing BDNF exons I, III, IV and the common exon IX in the hippocampus of pregnant rats and their embryos after Eth administration for 20 days. In pregnant rats (Figure 1), none of the Eth doses tested had statistically significant effects on the expression of BDNF exons I, III and IV compared with control rats. However, Eth at the doses of 0.25 and 2.5 g/kg·BW/day reduced the expression levels of the common exon IX, an effect that achieved significance (*p* < 0.05) at the Eth dose of 2.5 g/kg·BW/day.

**Table 1:** Experimental groups.

Treatment	Normal Saline	Ethanol 250 mg/kg·BW			Ethanol 630 mg/kg·BW			Ethanol 2500 mg/kg·BW		
	CT	Eth	Rsv mg/kg·BW		Eth	Rsv mg/kg·BW		Eth	Rsv mg/kg·BW	
	NS	Eth	60/Eth	120/Eth	Eth	60/Eth	120/Eth	Eth	60/Eth	120/Eth
Group	1	2	3	4	5	6	7	8	9	10

CT, control; Eth, ethanol; Rsv, resveratrol; NS, normal saline; BW, body weight.

This result obtained in pregnant rats is consistent with those previously reported in adult male rats, showing that chronic treatment of animals with Eth led to unchanged [53,54] or decreased [52,56,57] hippocampal levels of exon IX-containing BDNF transcripts. Tapia-Arancibia *et al.* [52] reported that BDNF mRNA levels were decreased when rats were exposed to chronic Eth, but the levels were increased 12 h after Eth withdrawal. However, other investigators have reported that acute doses of more than 5 g/kg Eth increased the expression level of the BDNF exon IX in male rats [58,59] or that of BDNF exons II, III and IV in the hippocampus of C57BL/6J mice [58]. Some of the inconsistencies in the results may be a result of the dose and route of Eth exposure or the timing of the measurements.

**Figure 2:**
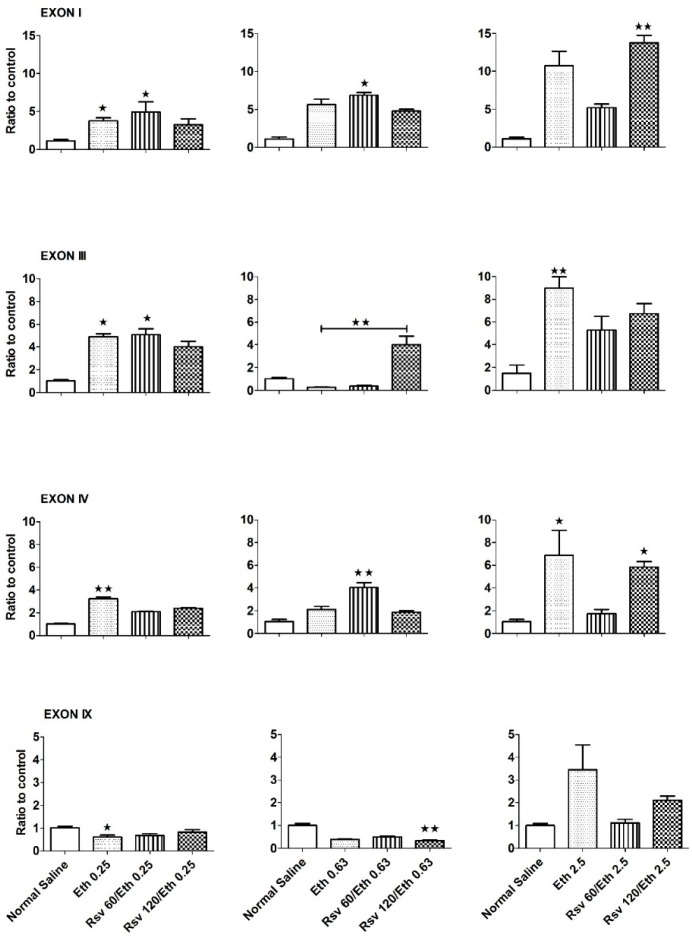
Differential effects of Eth and Rsv on the expression of BDNF exons in the hippocampus of embryonic rats. Eth (0.25 g/kg·BW/day) significantly increased expression of BDNF exons I, III and IV (*p* < 0.05 for exon I and III and *p* < 0.01 for exon IV) but decreased expression of BDNF exon IX compared to controls (*p* < 0.05). Eth (0.63 g/kg·BW/day) had no significant effect on the expression of these exons. Eth (2.5 g/kg·BW/day) increased expression of all BDNF exons but this increase was significant only for BDNF exons III and IV (*p* < 0.01 and *p* < 0.05, respectively). The combination of Eth (0.25 g/kg·BW/day) and Rsv (60 or 120 mg/kg·BW/day) had no significant effect on the expression of BDNF exon I, III and IV, but it reversed the decreased expression of BDNF exon IX induced by Eth (0.25 g/kg·BW/day). The combination of Rsv with Eth (0.63 g/kg·BW/day) caused increased expression of BDNF exons I, III and IV compared to the control group (*p* < 0.05 for exon I and *p* < 0.01 for exon III and IV), and for exon III, this increase was significant compared to the Eth group. This combination significantly decreased the expression of BDNF exon IX compared to the control group (*p* < 0.01). Combination of Rsv with Eth (2.5 g/kg·BW/day) significantly increased expression of BDNF exon I (*p* < 0.01) with no effect on the expression of other exons compared to the control group. The expression of BDNF transcripts containing exons I, III, IV and IX in the hippocampi of embryonic rats was tested using qRT-PCR. Pregnant rats were treated via oral gavage using either Eth alone or in combination with Rsv for 20 days, from day 1 to 20 of gestation. Another group received the same treatment except that they received normal saline only by gavage to serve as the control group (Table 1). Half of the hippocampi from each embryo of the four pregnant rats were pooled to make one sample (three samples in each group). β-actin expression was used to normalize gene expression in each sample to eliminate differences in RNA quality and amplification efficacy. Data are presented as the fold increase in expression in treatment groups compared to the control group, and are presented as the mean ± SEM. * *p* < 0.05; ** *p* < 0.01.

Eth produced a different pattern of effects in the hippocampus of embryonic rats (Figure 2). In this tissue, Eth at the dose of 0.25 g/kg·BW/day induced the expression of BDNF exons I, III and IV (*p* < 0.05 for exon I and III and *p* < 0.01 for exon IV), but decreased that of the common exon IX compared to controls (*p* < 0.05). Similarly, Eth at a dose of 2.5 g/kg·BW/day caused a significant increase in the expression of BDNF exons III and IV (*p* < 0.01 and 0.05, respectively). On the other hand, Eth at the dose of 0.63 g/kg·BW/day showed no significant effect on any of the exons. This type of effect (increased expression of BDNF exons III and IV in the Eth (0.25 g/kg·BW/day) group that was accompanied by a decrease in response to Eth (0.63 g/kg·BW/day) and followed by an increase in Eth (2.5 g/kg·BW/day)) had a U-shaped hormetic dose-response presentation. Eth is a compound for which a hormesis effect has been frequently reported [60,61]. However, to clarify this effect in embryos, further studies with additional doses are necessary.

### 2.2. Effects of Combined Eethanol and Rresveratrol on the Expression Pattern of Four Transcripts of the BDNF Gene in the Hippocampi of Adult Rats

To our knowledge, no data is available on the effects of Eth on the expression pattern of BDNF exons in the hippocampus of embryonic rats. However, similar to our data, Feng *et al.* [62], observed unchanged or reduced levels of total BDNF mRNA in rats that were prenatally (days 5–20) exposed to Eth but were sacrificed seven days after birth. In addition, rats exposed to Eth vapor for 3 h a day between postnatal days 10 and 15 and sacrificed at different days thereafter showed an age-related response to this treatment. Thus, pups that were decapitated at postnatal days 16 and 20 showed increased levels of exon IX containing BDNF mRNA in their hippocampus, in contrast to those decapitated at postnatal day 60 that showed a decreased level of the transcript [63]. These data suggest that the age of treatment and the length of Eth abstinence may have affected BDNF mRNA levels in the above-mentioned studies.

Results from several studies suggest that BDNF exon expression is differentially regulated through epigenetic changes in their individual promoters. This type of regulation has been shown for BDNF gene exons IV [64], I [65] and VI [66]. In addition, increased expression of individual promoters in BDNF exons has been accompanied by withdrawal [67,68] and/or addiction [69,70]. These findings encouraged investigators to study BDNF exon expression differentially as a potential therapeutic tool [71,72,73,74].

Several studies have reported neuroprotective effects of Rsv against neurocytotoxic effects of Eth [75,76,77]. Although the exact mechanism of this neuroprotection is not clear, it is hypothesized that decreased production of reactive oxygen species [78,79], upregulation of Sirtuin 1 and AMPK pathway [80,81] and suppression of NF-κB and AP-1 [36] may play a role in this effect. We have already reported that Rsv increases the expression of exon IX-containing BDNF transcripts in the hippocampus of male rats. This finding shows that increased expression of the BDNF gene may contribute in the neuroprotective effects of Rsv [38]. We extended our previous research to the effects of different doses of Eth alone and/or combined with Rsv on the expression pattern of various exons of the BDNF gene and its encoded protein, pro-BDNF, on the embryonic rats and their mothers.

As shown in Figure 1, pregnant rats exposed to Rsv (120 mg/kg·BW/day) in combination with Eth (0.25 g/kg·BW/day) showed increased expression levels of BDNF gene exons I and III (*p* < 0.05). In these rats, the increase observed in the level of BDNF exon IV was only significant compared to that of animals that received a 0.25 g/kg·BW/day dose of Eth alone (*p* < 0.05). When combined with Eth (0.63 and/or 2.5 g/kg·BW/day), neither 60 nor 120 mg/kg·BW/day Rsv had significant effects on the expression of BDNF transcripts containing exon I, III or IV compared to the normal saline group. However, Rsv (120 mg/kg·BW/day) caused a significant increase in the expression of BDNF transcripts containing exon III compared to the Eth (0.63 mg/kg·BW/day) group (*p* < 0.05). In addition, Rsv at doses of 60 and 120 mg/kg·BW/day reversed the decreasing effects of Eth (2.5 g/kg·BW/day) on BDNF exon IX expression.

Taken together, when combined with a low dose (0.25 g/kg·BW/day) of Eth, Rsv tended to increase the expression of the BDNF exons tested in this study. This observation is consistent with our previous report on male rats [38] and with other published data on the effects of Rsv on the expression of BDNF *in vivo* or *in vitro* under a cytotoxic stimuli such as depression [82,83], Eth [77] or hypoxia [84]. In contrast to these findings, Park *et al.* [85] showed a downregulating effect of Rsv on the expression of BDNF mRNA in both animal and cellular models. We are the first to report the effect of Rsv treatment on the expression pattern of the BDNF gene.

### 2.3. Effects of Combined Eethanol and Rresveratrol on the Expression Pattern of Four BDNF Gene Transcripts in the Embryonic Rat Hippocampus

Protective effects of Rsv against Eth-induced cytotoxicity are reported in the CNS during early postnatal life [76,86,87]. Therefore, we analyzed the effect of combined Rsv and Eth on the expression pattern of BDNF exons in the hippocampus of embryonic rats.

As shown in Figure 2, co-administration of Eth (0.25 g/kg·BW/day) with Rsv did not produce any significant changes in the above-mentioned effects of Eth alone. Combined treatment of Eth (0.63 g/kg·BW/day) with Rsv (60 mg/kg·BW/day) led to a significant increase in the expression of BDNF exons I and IV compared with those of control embryos (*p* < 0.05 and *p* < 0.01, respectively, Kruskal-Wallis/Dunn’s test). On the other hand, administration of Eth and Rsv (120 mg/kg·BW/day) caused a significant decrease in the expression level of BDNF exon IX (*p* < 0.01) compared with the normal saline group and it also caused a significant increase in the expression of exon III compared with the Eth (0.63 g/kg·BW/day) group (*p* < 0.01). Embryonic rats exposed to a combination of Eth (2.5 g/kg·BW/day) and Rsv (120 mg/kg·BW/day) showed an increase in the expression of all BDNF exons when compared to their control counterparts, but this increase was statistically significant only for exons I and IV (*p* < 0.01 and *p* < 0.05, respectively). Thus, in embryos, Eth showed a tendency to increase the expression of BDNF exons I, III and IV but not the common exon IX. The reason for this exception may be a reduction in the expression levels of other BDNF mRNA variants. These variants are transcripts that contain BDNF exons and they were not tested in this study.

### 2.4. Effects of Eethanol and Rresveratrol on the Levels of Pro-BDNF Protein in the Hippocampus of Pregnant Rats and Their Embryos

As stated above, all 11 BDNF transcripts are translated into one single pro-BDNF protein. Although pro-BDNF is a precursor of BDNF, hippocampal neurons are reported to secrete pro-BDNF rather than mature BDNF in response to activity [23]. There is much evidence on the opposing effects of pro-BDNF and BDNF on neuronal cell survival that are exerted through separate receptors [24]. Thus, Woo *et al.* [25] suggested that the pro-BDNF effect on p75NT receptors is a mechanism of induction of cell death in the hippocampus.

**Figure 3:**
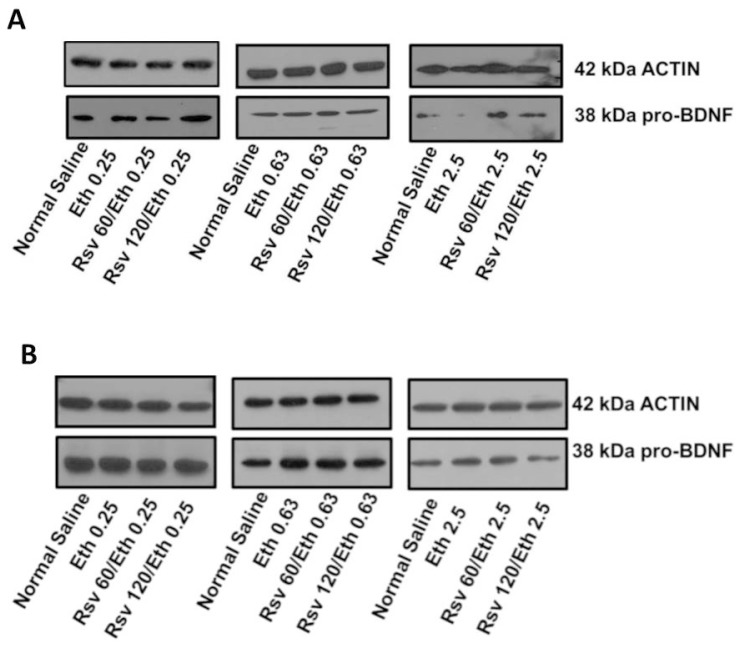
Eth alone or in combination with Rsv affects the pro-BDNF protein level in pregnant rats but not in the embryos. (**A**) Higher doses of Eth decreased pro-BDNF protein levels in the hippocampus of pregnant rats. Rsv in combination with the lower doses of Eth increased pro-BDNF protein levels in these tissues; (**B**) In the tissue extracts from embryos, Eth alone or in combination with Rsv had no effect on the pro-BDNF protein level. Immunoblots were arranged to identify pre-mature (pro-) BDNF in tissue extracts from hippocampi of pregnant and embryonic rats. β-actin was used as a loading control. Each blot is representative of three replicates. Antibodies against BDNF and β-actin detected proteins bands of approximately 38 and 42 kDa for pro-BDNF and β-actin, respectively.

Western blotting was performed to analyze levels of pro-BDNF protein in the hippocampi of pregnant and embryonic rats in response to Eth alone or in combination with Rsv (Figure 3). In adult rats (Figure 3A), Eth (0.25 g/kg·BW/day) had no significant effect on the pro-BDNF level, while in combination with Rsv (120 mg/kg·BW/day), it caused a small increase in the pro-BDNF level. This dose of Eth either alone or in combination with Rsv did not affect pro-BDNF levels in the hippocampus of rat embryos. Eth (0.63 g/kg·BW/day) either alone or combined with Rsv caused no significant changes in pro-BDNF protein levels in pregnant and/or embryonic rats. Eth (2.5 g/kg·BW/day) produced a small but reproducible decrease in the pro-BDNF protein level in the hippocampi of pregnant rats, an effect that was reversed by both doses of Rsv tested here. This result is consistent with findings of other investigators on the decreasing effects of Eth on BDNF in the hippocampus [51] and cortex [88] in male rats.

The decreasing effect of Eth (2.5 g/kg·BW/day) was not observed in specimens from embryos exposed to Eth (2.5 g/kg·BW/day) alone or in combination with any of the Rsv doses of 60 or 120 mg/kg·BW/day (Figure 3B).

Pups that had been prenatally exposed to Eth revealed unchanged levels of BDNF protein in their postnatal hippocampus [9,62]. Feng *et al.* [62] also showed that increasing the dose of Eth from 1 to 3 g/kg/day reduced the BDNF protein level, as seen in our data. Unexpectedly, scientists who used higher doses of Eth (more than 5 g/kg/day) reported elevated BDNF protein levels in the hippocampus and frontal cortex of neonatal rats [11,89]. Another study reported an increased BDNF protein level in the hippocampus of pups decapitated at postnatal day (PD) 10, but the BDNF level returned to that of controls by PD 21 [90]. Therefore, the BDNF protein level appears to depend on the Eth dose used and on the developmental stage of the rat. Controversial data is reported on the effects of Rsv on the BDNF protein. While results of a few studies show that Rsv (20–80 mg/kg intraperitoneal) has an inducing effect on BDNF levels in the prefrontal cortex and hippocampus of mice [91,92], Park *et al.* [92], reported reduced level of BDNF protein in the hippocampus of mice treated with low doses of Rsv (1–10 mg/kg i.p.).

Comparison of the effect of Eth alone or in combination with Rsv on the level of pro-BDNF protein between adult and embryo hippocampus tissue show that significant changes in the level of BDNF exons in response to these treatments were accompanied by changes in the pro-BDNF protein levels in adult rats, while this association was not observed in embryos. This suggests that post-transcriptional regulation (regulation at the level of translation) may be more important and exerts more severe effects in embryos.

Future studies will involve investigating the epigenetic changes that Eth and Rsv exert in the individual promoters of the BDNF gene.

In conclusion, our data summarized in Table 2, show that although a low dose of Eth has no significant effect on the expression pattern of BDNF in the adult rats, it can affect the pattern in the embryo hippocampus. When combined with a low dose of Eth, Rsv tended to increase the expression of the BDNF exons in pregnant rats tested in this study. In embryos, however, Eth alone showed a tendency to increase the expression of BDNF exons I, III and IV but not the common exon IX. These observations emphasize that the effects of Eth on the BDNF expression in the hippocampus depends on the dose of Eth administered and on the developmental stage of the animals. Significant changes in the expression level of BDNF exons in response to treatments used in this study were accompanied by changes in the pro-BDNF protein levels in adult but not embryonic rats.

**Table 2:** Effect of three Eth doses alone or in combination with Rsv (60 and/or 120 mg/kg·BW/day) on BDNF mRNA and protein levels in the hippocampus of adult and embryonic rats.

Treatment	Adult					Embryo				
	mRNA				Protein	mRNA				Protein
	Exon I	Exon III	Exon IV	Exon IX	Pro-BDNF	Exon I	Exon III	Exon IV	Exon IX	Pro-BDNF
Eth 0.25	NS	NS	NS	NS	NS	↑	↑	↑	↓	NS
Rsv 60/Eth 0.25	NS	NS	NS	NS	NS	↑	↑	NS	NS	NS
Rsv 120/Eth 0.25	↑	↑	NS	NS	↑	NS	NS	NS	NS	NS
Eth 0.63	NS	NS	NS	NS	NS	NS	NS	NS	NS	NS
Rsv 60/Eth 0.63	NS	NS	NS	NS	NS	↑	NS	↑	NS	NS
Rsv 120/Eth 0.63	NS	NS	NS	NS	NS	NS	NS	NS	↓	NS
Eth 2.5	NS	NS	NS	↓	↑	NS	↑	↑	NS	NS
Rsv 60/Eth2.5	NS	NS	NS	NS	NS	NS	NS	NS	NS	NS
Rsv 120/Eth 2.5	NS	NS	NS	NS	NS	↑	NS	↑	NS	NS

NS, non-significant changes compared to the normal saline group; ↑, significant increase compare to the normal saline group; ↓,significant decrease compared to the normal saline group.

## 3. Experimental Section

### 3.1. Subjects and Experimental Design

Female Sprague-Dawley rats, weighting 200–250 g (*n* = 120), were provided by the Laboratory Animal Center of Shiraz University of Medical Sciences, Shiraz, Iran. Rats were mated and detection of vaginal plaque was considered as the first day of pregnancy. Pregnant rats (300–350 g) were divided into 10 groups of 12 as shown in Table 1.

Rats in the control group received normal saline by gavage and rats in the Eth groups received 0.25, 0.63 or 2.5 g/kg·BW/day Eth (Merck, Germany) also by gavage. To obtain the desired Eth concentration, absolute Eth was diluted with normal saline. Rats in combination treatment groups were treated with either 60 or 120 mg/kg·BW/day Rsv (98% purity; Biotivia, New York, NY, USA) in combination with one of the 3 Eth doses. Rats were housed 4 to a cage in transparent cages (59 × 38 × 20 cm) and received water and food ad libitum under a 12 h light/dark cycle. Treatments were performed by daily gavage at 9 am from, day 1 to 20 of pregnancy. Rats were decapitated after CO_2_ inhalation and hippocampal tissues were isolated from both mothers and embryos 24 h after the last gavage. Isolated hippocampi of four female rats and/or isolated hippocampi of embryos of four mothers in each group were combined to make one sample. The right hippocampi of adult rats were collected separately and allocated for mRNA analysis, while the left half were allocated for protein analysis. Hippocampi of half of embryos from each dam were pooled for mRNA analysis and the remaining numbers were collected for the protein assay. All experimental protocols were performed in accordance with the National Institutes of Health Guide for Care and Use of Laboratory Animals and were approved by the Medical and Research Ethics Committee of the Shiraz University of Medical Sciences, Shiraz, Iran.

### 3.2. Real-Time Quantitative PCR

Immediately after dissection, samples allocated for mRNA analysis were added to Biozol reagent (BSC51M1, BioFlux, Tokyo, Japan) and stored at −80 °C until RNA extraction. RNA was extracted using the Biozol kit (BSC51M1, BioFlux, Tokyo, Japan), according to the manufacturer’s protocol, and analyzed by Nano-drop to define their concentration and purity. The denaturing gel electrophoresis method was used to test the RNA integrity [93]. RNA was treated with DNase I (EN0521, Fermentas, Opelstrasse, Germany) to eliminate any DNA contamination. cDNAs were synthesized with 5 μg of RNA and 1 μL of oligo dTs using RevertAid First Strand cDNA Synthesis Kit (K1621, Fermentas, GmbH, St. Leon-Rot, Germany). All procedures were based on the manufacturer’s protocol.

Quantitative RT-PCR was performed, as previously described [94]. Briefly, a mixture containing 10 µL SYBR Premix Ex Taq II (RR820L, Tli RNaseH Plus, TaKaRa, Kyoto, Japan), 0.08 µL ROX reference dye, 0.2 µM of each of the primers and 100 ng cDNA were prepared and the gene segments of interest, and were amplified using a 7500 real-time PCR system (Applied Biosystem, Foster City, CA, USA). The following protocol was used for all gene amplification: initial denaturation at 95 °C for 30 s, 40 cycles of 95 °C for 5 s and annealing and elongation at 60 °C for 30 s. Primer sequences are shown in Table 3. The three reference genes, hypoxanthine guanine phosphoribosyl transferase (HPRT), glyceraldehyde 3-phosphate dehydrogenase (GAPDH) and β-actin were tested for expression stability and β-actin was selected as the most stable gene to be used for normalization of the data. All PCR reactions were run in duplicate. The ΔΔ*C*_T_ method was used to calculate the ratio of BDNF exon expression level [95]. The quality and accuracy of the PCR products were tested using electrophoresis on 1.6% agarose gels.

**Table 3:** Primer sequences used for real-time PCR to amplify genes of interest.

Gene Name	Primer Forward (5′ to 3′)	Primer Reverse (5′ to 3′)	Reference
BDNF EXON I	TGTTGGGGAGACGAGATTTT	CGTGGACGTTTGCTTCTTTC	[96]
BDNF EXON III	CTGAGACTGCGCTCCACTC	GTGGACGTTTGCTTCTTTCA	[96]
BDNF EXON IV	GAGCAGCTGCCTTGATGTTT	GTGGACGTTTGCTTCTTTCA	[96]
BDNF EXON IX	GTGACAGTATTAGCGAGTGGG	GGGTAGTTCGGCATTGC	[94]
β-actin	CCACACCCGCCACCAGTTCG	CTAGGGCGGCCCACGATGGA	[94]
HPRT	CCCAGCGTCGTGATTAGTGA	TGGCCTCCCATCTCCTTCAT	*
GAPDH	CGTGATCGAGGGCTGTTGG	CTGCTTCAGTTGGCCTTTCG	[97]

*, primer designed by author.

### 3.4. Western Blotting

The samples allocated for protein analysis were transferred to liquid nitrogen immediately after isolation and stored at −80 °C until analysis. The extraction buffer used to extract proteins contained a protease inhibitor cocktail (P8340, Sigma-Aldrich, St. Louis, MO, USA) and NP40 lysate buffer. As previously described [98,99], Western blotting was performed by homogenizing the samples followed by 3 replicates of sonication for 5 s. This was followed by centrifugation of samples for 10 min at 10,000× *g* at 4 °C, and the supernatant was collected. The Bradford method was used for measurement of protein concentrations and 80 µg protein from each sample was separated using 15% sodium dodecyl sulfate-polyacrylamide gel electrophoresis (SDS-PAGE) and the PageRuler^TM^ Plus prestained protein ladder (26619, Fermentase, Rockford, IL, USA) was used to define the weight of separated bands. Separated samples were transferred to nitrocellulose membrane at 100 V for 90 min (Mini Trans-Blot Cell, Bio-Rad, Berkeley, CA, USA) and non-specific binding sites were blocked by soaking membranes in 5% skimmed milk in 0.2% TBST for 1 h at room temperature. We then incubated membranes overnight at 4 °C with either a 1:500 titer of a polyclonal rabbit anti-BDNF antibody (sc-546, SantaCruz Biotechnology, Santa Cruz, CA, USA) or a 1:1000 dilution of the anti β-actin antibody (ab1801, Abcam, Burlingame, CA, USA) in 1% skimmed milk −0.2% TBST. β-actin was used as internal control. Membranes were then rinsed 3 times for 20 min each using 0.2% TBST and treated with the secondary antibody (1:15,000 goat anti-rabbit IgG: HRP antibody, Aviva System Biology, San Diego, CA, USA) for 1 h at room temperature. This was followed by rinsing 3 times with 0.2% TBST for 20 min and by development of membranes using a home-made enhanced chemiluminescence (ECL) (250 mM 3-aminophthalhydrazide (Luminol) (123072, Sigma-Aldrich, St. Louis, MO, USA), 90 mM p-Coumaric acid (H23201, Sigma-Aldrich, St. Louis, MO, USA), 30% H_2_O_2_ and 100 mM Tris, pH 8.6) and 13 × 18 AGFA X-ray films. AlphaEaseFC software (Alpha Innotech, San Leandro, CA, USA) was used to quantify the band intensity.

### 3.5. Data Analysis

The software package Prism (GraphPad Prism version 5, GraphPad Software, San Diego, CA, USA) was used for statistical analysis and results were expressed as the mean ± SEM. A nonparametric analysis of variance (Kruskal-Wallis test) was performed to detect any differences between groups and Dunn’s multiple comparison test was conducted as the post-test whenever significant differences were detected between groups. *p* < 0.05 was considered to be statistically significant.
